# Dexmedetomidine Attenuates LPS-Induced Monocyte-Endothelial Adherence via Inhibiting Cx43/PKC-*α*/NOX2/ROS Signaling Pathway in Monocytes

**DOI:** 10.1155/2020/2930463

**Published:** 2020-07-19

**Authors:** Yunfei Chai, Zhongming Cao, Runying Yu, Yong Liu, Dongdong Yuan, Liming Lei

**Affiliations:** ^1^Anesthesiology Department of Guangdong Cardiovascular Institute, Guangdong Provincial People's Hospital, Guangdong Academy of Medical Sciences, Guangzhou, Guangdong, China; ^2^Operating Centre of Guangdong Provincial People's Hospital, Guangdong Academy of Medical Sciences, Guangzhou, Guangdong, China; ^3^Cardiology Department of Guangdong Cardiovascular Institute, Guangdong Provincial People's Hospital, Guangdong Academy of Medical Sciences, Guangzhou, Guangdong, China; ^4^Department of Anesthesiology, The Third Affiliated Hospital of Sun Yat-sen University, Tianhe Road Guangzhou, Guangdong, China; ^5^Department of Intensive Care Unit of Cardiovascular Surgery, Guangdong Cardiovascular Institute, Guangdong Provincial People's Hospital, Guangdong Academy of Medical Sciences, Laboratory of South China Structural Heart Disease, Guangzhou, Guangdong, China

## Abstract

Dexmedetomidine is widely used for sedating patients in operation rooms or intensive care units. Its protective functions against oxidative stress, inflammation reaction, and apoptosis have been widely reported. In present study, we explored the effects of dexmedetomidine on monocyte-endothelial adherence. We built lipopolysaccharide- (LPS-) induced monocyte-endothelial adherence models with U937 monocytes and human umbilical vein endothelial cells (HUVECs) and observed the effects of dexmedetomidine on U937-HUVEC adhesion. Specific siRNA was designed to knock-down Connexin43 (Cx43) expression in U937 monocytes. Gö6976, GSK2795039, and NAC were used to inhibit PKC-*α*, NOX2, and ROS, respectively. Then, we detected whether dexmedetomidine could downregulate Cx43 expression and its downstream PKC-*α*/NOX2/ROS signaling pathway activation and ultimately result in the decrease of U937-HUVEC adhesion. The results showed that dexmedetomidine, at its clinically relevant concentrations (0.1 nM and 1 nM), could inhibit adhesion of molecule expression (VLA-4 and LFA-1) and U937-HUVEC adhesion. Simultaneously, it also attenuated Cx43 expression in U937 monocytes. With the downregulation of Cx43 expression, the activity of PKC-*α* and its related NOX2/ROS signaling pathway were reduced. Inhibiting PKC-*α*/NOX2/ROS signaling pathway with Gö6976, GSK2795039, and NAC, respectively, VLA-4, LFA-1 expression, and U937-HUVEC adhesion were all decreased. In summary, we concluded that dexmedetomidine, at its clinically relevant concentrations (0.1 nM and 1 nM), decreased Cx43 expression in U937 monocytes and PKC-*α* associated with carboxyl-terminal domain of Cx43 protein. With the downregulation of PKC-*α*, the NOX2/ROS signaling pathway was inhibited, resulting in the decrease of VLA-4 and LFA-1 expression. Ultimately, U937-HUVEC adhesion was reduced.

## 1. Introduction

The recruitment of circulating monocytes to inflamed tissues is one of the most important characters of acute and chronic inflammatory responses. The migration of monocytes involves sequential molecular interactions with endothelial cells, known as the adhesion cascade, in which firm monocyte-endothelial adherence is the fundamental step [1, 2]. There are lots of risk factors that can increase monocyte-endothelial adherence, the most important one of which is just LPS, an outer membrane component of Gram-negative bacteria [1, 3]. LPS directly activates monocytes, resulting in its adherence to endothelial cells or the extracellular matrix [4]. The levels of LPS are generally elevated in patients undergoing major surgeries or remaining in the intensive care units [5, 6]. In addition, long-term supine position also leads monocyte-endothelial adherence to become easier. Therefore, monocyte-endothelial adherence and its related inflammatory damage are more likely to occur in such critical patients.

We notice that dexmedetomidine, as a highly selective alpha-2 adrenoceptor agonist with sedative, analgesic, and anesthetic effects, is widely used for sedating patients in operation rooms or intensive care units [7, 8]. Its multifaceted protective functions for the heart, nerve, kidney, and liver have been widely reported [7]. Our previous study also showed that dexmedetomidine attenuated monocyte-endothelial adherence when acting on vascular endothelial cells [7]; however, the effects of dexmedetomidine on monocytes were still poorly understood. Therefore, in this study, we explored the effects of dexmedetomidine on monocytes and LPS-induced monocyte-endothelial adherence.

Connexins are expressed in almost all human organs and tissues. Cx37, Cx40, and Cx43 are predominantly expressed in cardiovascular system [9, 10]. Multiple reports clarify the importance of Cx43 on monocyte-endothelial adherence [10, 11]. Although the change of Cx43 expression on cell membranes can regulate a series of signaling pathways, the Cx43-related signal transduction from cell membranes to cytoplasm is still not clear. According to the analysis of the transmembrane structure of Cx43 protein, we notice that its carboxyl-terminal domain can interact with some cellular signaling pathways, the most important one of which is just PKC. PKC activation has been identified to play an important part in inflammation and oxidative stress [12]. This kind of interaction provides the possibility that Cx43 expression on cell membrane influences other signaling pathways in cytoplasm. The cross talk between Cx43 and PKC is the basis of Cx43 regulating downstream signaling pathways in cytoplasm [9].

As a kind of protein kinase, PKC triggers the activation of NADPH oxidase (NOX), in which NOX2 is currently accepted as the main source of ROS [13, 14]. ROS-mediated oxidative stress is thought to be closely related to all known cardiovascular diseases [15, 16]. It can activate NF-*κ*B, JNK/SAPK, and p38 MAPK signaling pathways, resulting in the upregulation of adhesion of molecule expression significantly [17, 18]. Thus, we speculate that dexmedetomidine decreases Cx43 expression on cell membrane and PKC-*α* associated with carboxyl-terminal domain of Cx43 protein. With the downregulation of PKC-*α*, the NOX2/ROS signaling pathway is inhibited, resulting in the decrease of adhesion of molecule expression and monocyte-endothelial adherence.

## 2. Materials and Methods

### 2.1. Cell Culture

The present study protocol conforms to the ethical guidelines of the 1975 Declaration of Helsinki with the approval of the Institutional Medical Ethics Committee of the Third Affiliated Hospital of Sun Yat-sen University. The cell lines of U937 monocytes and HUVECs are both purchased from the American Type Culture Collection (Manassas, VA, USA). U937 monocytes are cultured in RPMI1640 medium (Invitrogen, Carlsbad, CA, USA) with 20% fetal bovine serum (Invitrogen). HUVECs are cultured with human endothelial SFM (Invitrogen) with 20% fetal bovine serum (Invitrogen), 100 *μ*g/ml heparin (Sigma-Aldrich, St. Louis, MO, USA), and 200 *μ*g/ml endothelial cell growth supplement (Becton, Dickinson and Company, Frankin Lakes, NJ, USA). The 3 to 5 passages are used for experiments. Both U937 monocytes and HUVECs are cultured in a 5% CO_2_ incubator at 37°C and 90% humidity (Thermo Fisher Scientific, Waltham, MA, USA).

### 2.2. Cell Treatments

Dexmedetomidine is obtained from Hengrui Medicine (Jiangsu, China). U937 monocytes are pretreated with dexmedetomidine (0.1 nM and 1 nM) for 24 hours before other experiments. NAC (Sigma-Aldrich, 10 mM, 1 hour) is used to scavenge ROS. GSK2795039 (MedChemExpress, NJ, USA, 25 *μ*M, 24 hours) is used to inhibit NOX2. Gö6976 (Cell Signaling Technology, Danvers, MA, USA, 10 *μ*M, 24 hours) is used to inhibit PKC-*α*.

### 2.3. Adhesion Assay

Before adhesion assay, U937 monocytes are firstly pretreated with LPS (1 *μ*g/ml) for 24 hours. Then, the cells are labeled with 5 *μ*M calcein-acetoxymethyl ester (Invitrogen) and cultured in the incubator. Thirty minutes later, the labeled U937 monocytes are washed twice with PBS (Invitrogen) and resuspended in the medium without serum. After that, they are added onto the confluent monolayers of HUVECs. The plates are put back into the incubator for one-hour incubation. When the incubation is finished, the plates are rinsed twice slightly with medium. Adherent U937 monocytes are left and remain on the confluent monolayers of HUVECs. We count the adherent U937 monocytes with a fluorescence microscope (Olympus IX71, Tokyo, Japan). For each well, 8 different visual fields (×200) in the middle of the dish are chosen for analysis.

### 2.4. Cx43 Knock-Down with Specific siRNA

We design specific siRNA targeting to Cx43 expression in U937 monocytes (siRNA-Cx43: GCTGGTTACTGGTGACAGA) and a nonspecific, control siRNA (NC as shown in the figures). Transfection into U937 monocytes is carried out by using Lipofectamine 2000 (Invitrogen, Carlsbad, CA, USA) according to the manufacturer's instruction. Western blotting is used to test the efficiency of knock-down.

### 2.5. Western Blotting

The expressions of VLA-4, LFA-1, NOX2, PKC-*α*, and Cx43 are detected by western blotting, followed the standard procedures as described [12]. Briefly, U937 monocytes are washed with cold PBS twice and harvested in lysis buffer (Bio-Rad, Hercules, CA), sonicated, and centrifuged for 30 min at 4°C. The Pierce™ BCA Protein Assay Kit is used to quantify the protein samples (Thermo Fisher Scientific, Inc., Waltham, MA, USA). Then, 30 *μ*g of each protein sample is added into SDS-PAGE and subsequently transferred onto a polyvinylidene fluoride membrane. These membranes are blocked for 1 hour with 5% milk (nonfat-dried milk bovin/TBST solution: 5 g/100 ml; Sigma-Aldrich) at room temperature. Different primary antibodies are used to detect protein expression. The dilution of primary antibodies are as follows: anti-Cx43 (1 : 4000, Sigma-Aldrich); anti-VLA-4 and LFA-1 (1 : 100, Santa Cruz Biotechnology, Santa Cruz, CA, USA); anti-NOX2 (1 : 1000, Thermo Fisher Scientific); anti-PKC-*α* (1 : 1000, Thermo Fisher Scientific); and *β*-actin (1 : 5000, Thermo Fisher Scientific). Protein band sizes are valued by the Alpha View software (version number: 2.2.14407, Protein Simple, Santa Clara, CA, USA).

### 2.6. ROS Detection

The level of ROS is detected with the ROS assay kit (6-carboxy-2′-7′-dichlorodihydrofluorescein diacetate (DCFHDA), Thermo Fisher Scientific). Fluorescence can be monitored using a flow cytometer. The fluorescence intensity of each group is calculated following the manufacturer's instruction.

### 2.7. Statistical Analysis

Statistical analysis is performed by using the SPSS 15.0 software (SPSS, Inc., Chicago, IL, USA). Multiple comparisons among groups are analyzed using repeated measures one-way ANOVA, followed by Tukey's post hoc comparisons.

## 3. Results

### 3.1. Dexmedetomidine Attenuated U937-HUVEC Adhesion and the Expression of VLA-4 and LFA-1 in U937 Monocytes

**Figure 1:**
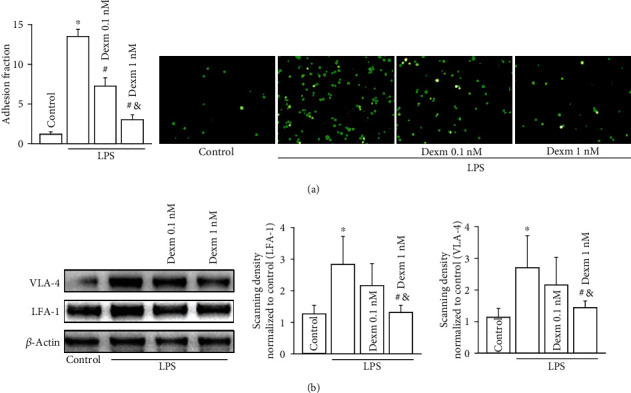
Dexmedetomidine attenuates U937-HUVEC adhesion and inhibits VAL-4 and LFA-1 expression in U937 monocytes. (a) Effects of dexmedetomidine on U937-HUVEC adhesion when dexmedetomidine acts on U937 monocytes (*n* = 5, ∗*P* < 0.05 vs. control; ^#^*P* < 0.05 vs. the LPS group; ^&^*P* < 0.05 vs. the 0.1 nM dexmedetomidine group). (b) Effects of dexmedetomidine on VLA-4 and LFA-1 expressions in U937 monocytes (*n* = 5, ∗*P* < 0.05 vs. control; ^#^*P* < 0.05 vs. the LPS group; ^&^*P* < 0.05 vs. the 0.1 nM dexmedetomidine group).

In our previous study, we found that dexmedetomidine at its clinically relevant concentrations (0.1 nM and 1 nM) [19, 20] could attenuate monocyte-endothelial adherence via inhibiting Cx43 on vascular endothelial cells [7]. Therefore, in present investigation, we observed the effects of dexmedetomidine on monocytes and monocyte-endothelial adherence. The results showed that when U937 monocytes were exposed to LPS, U937-HUVEC adhesion were increased significantly, which could be attenuated by dexmedetomidine (0.1 nM and 1 nM for 24 hours) application (Figure 1(a)). Meanwhile, the ligands of VCAM-1 and ICAM-1 expressed in U937 monocytes, VLA-4, and LFA-1 were also downregulated by dexmedetomidine (Figure 1(b)).

### 3.2. Dexmedetomidine Attenuated U937-HUVEC Adhesion and Adhesion of Molecule Expression in U937 Monocytes via Inhibiting NOX2-Mediated ROS Production

**Figure 2:**
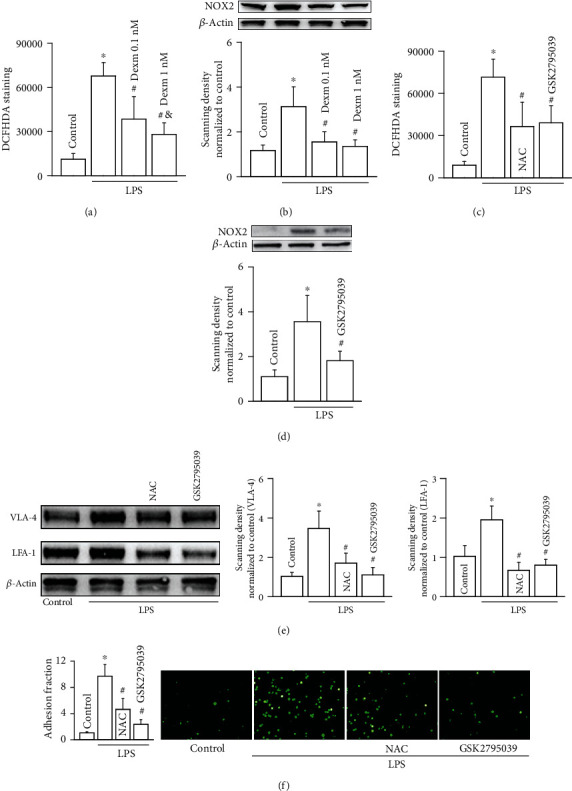
Dexmedetomidine attenuates U937-HUVEC adhesion and inhibits VAL-4 and LFA-1 expressions in U937 monocytes via downregulating the NOX2/ROS signaling pathway. (a) Effects of dexmedetomidine on the level of ROS in U937 monocytes (*n* = 5, ∗*P* < 0.05 vs. control; ^#^*P* < 0.05 vs. the LPS group; ^&^*P* < 0.05 vs. the 0.1 nM dexmedetomidine group). (b) Effects of dexmedetomidine on NOX2 expression in U937 monocytes (*n* = 5, ∗*P* < 0.05 vs. control; ^#^*P* < 0.05 vs. the LPS group). (c) Effects of NAC and GSK2795039 on the level of ROS in U937 monocytes (*n* = 5, ∗*P* < 0.05 vs. control; ^#^*P* < 0.05 vs. the LPS group). (d) Effects of GSK2795039 on NOX2 expression in U937 monocytes (*n* = 5, ∗*P* < 0.05 vs. control; ^#^*P* < 0.05 vs. the LPS group). (e) Effects of NAC and GSK2795039 on VLA-4 and LFA-1 expressions in U937 monocytes (*n* = 4, ∗*P* < 0.05 vs. control; ^#^*P* < 0.05 vs. the LPS group). (f) Effects of NAC and GSK2795039 on VLA-4 and LFA-1 expressions in U937 monocytes (*n* = 5, ∗*P* < 0.05 vs. control; ^#^*P* < 0.05 vs. the LPS group) and effects of NAC and GSK2795039 on U937-HUVEC adhesion (*n* = 5, ∗*P* < 0.05 vs. control; ^#^*P* < 0.05 vs. the LPS group).

NOX2-induced ROS generation is always considered to play an important part in monocyte-endothelial adherence [21, 22]. Thus, we detected the effects of dexmedetomidine (0.1 nM and 1 nM for 24 hours) on NOX2/ROS signaling pathway in the present study. Figures 2(a) and 2(b) demonstrate that LPS could activate NOX2/ROS signaling pathway in U937 monocytes, manifested as ROS production and NOX2 expression increase, while dexmedetomidine inhibited it effectively. NAC (the scavenger of ROS) and GSK2795039 (the inhibitor of NOX2) attenuated ROS generation and NOX2 expression in U937 monocytes effectively (Figures 2(c) and 2(d)), and more importantly, both of them decreased LPS-induced VLA-4 and LFA-1 expression increase in U937 monocytes, as well as U937-HUVEC adhesion (Figures 2(e) and 2(f)). These results demonstrated that dexmedetomidine (0.1 nM and 1 nM for 24 hours) could decrease LPS-induced adhesion of molecule expression (VLA-4 and LFA-1) and U937-HUVEC adhesion via inhibiting NOX2/ROS signaling pathway in U937 monocytes.

### 3.3. Dexmedetomidine Inhibited LPS-Induced NOX2/ROS Signaling Pathway Activation via Attenuating PKC-*α* in U937 Monocytes

**Figure 3:**
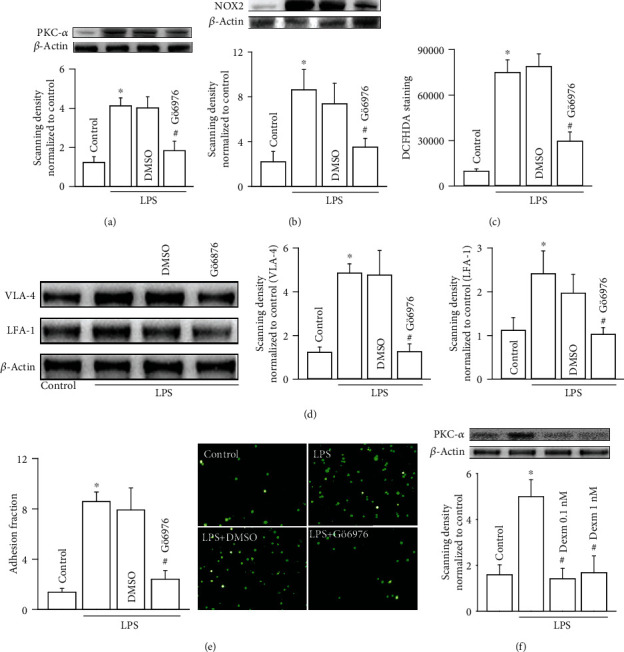
In U937 monocytes, inhibiting PKC-*α* with Gö6976 attenuates NOX2/ROS signaling pathway, and its downstream VAL-4 and LFA-1 expressions, ultimately resulting in U937-HUVEC adhesion decrease. (a) Gö6976 reduces PKC-*α* expression in U937 monocytes (*n* = 5, ∗*P* < 0.05 vs. control; ^#^*P* < 0.05 vs. the LPS group). (b) Effects of Gö6976 on NOX2 expression in U937 monocytes (*n* = 5, ∗*P* < 0.05 vs. control; ^#^*P* < 0.05 vs. the LPS group). (c) Effects of Gö6976 on the level of ROS in U937 monocytes (*n* = 5, ∗*P* < 0.05 vs. control; ^#^*P* < 0.05 vs. the LPS group). (d) Effects of Gö6976 on VLA-4 and LFA-1 expressions in U937 monocytes (*n* = 4, ∗*P* < 0.05 vs. control; ^#^*P* < 0.05 vs. the LPS group). (e) Effects of Gö6976 on U937-HUVEC adhesion (*n* = 5, ∗*P* < 0.05 vs. control; ^#^*P* < 0.05 vs. the LPS group). (f) Effects of dexmedetomidine on PKC-*α* expression in U937 monocytes (*n* = 5, ∗*P* < 0.05 vs. control; ^#^*P* < 0.05 vs. the LPS group).

PKC-*α* expression in U937 monocytes was attenuated by the inhibitor of PKC-*α*, Gö6976 (Figure 3(a)), and simultaneously, NOX2 expression and ROS generation were both downregulated (Figures 3(b) and 3(c)). Subsequently, the downstream VLA-4 and LFA-1 expressions were decreased and U937-HUVEC adhesion were reduced because of PKC-*α* inhibition (Figures 3(d) and 3(e)). In Figure 3(f), dexmedetomidine (0.1 nM and 1 nM for 24 hours) attenuated LPS-induced PKC-*α* increase in U937 monocytes obviously. From all of the above, we speculated that in U937 monocytes, LPS exposure resulted in PKC-*α*-mediated NOX2/ROS signaling pathway activation, ROS generation becoming more, upregulation of adhesion of molecule expression (VLA-4 and LFA-1), and ultimately, U937-HUVEC adhesion increasing. Dexmedetomidine application could alleviate LPS-induced U937-HUVEC adhesion via inhibiting PKC-*α* and its downstream signaling pathway.

### 3.4. Dexmedetomidine Inhibited LPS-Induced Cx43 Increase in U937 Monocytes, which Could Interact with PKC-*α*

**Figure 4:**
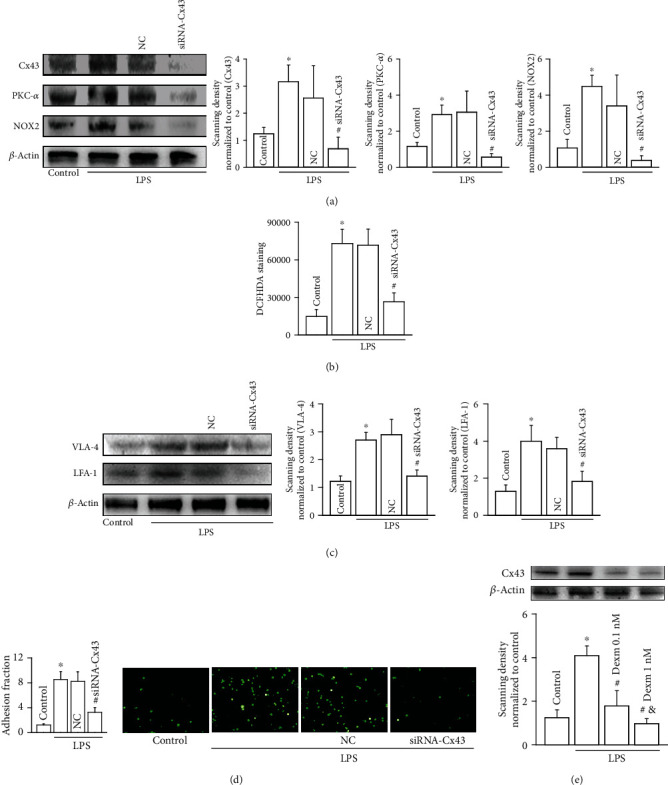
Dexmedetomidine attenuates Cx43 expression in U937 monocytes. With the downregulation of Cx43 expression, the PKC-*α*/NOX2/ROS signaling pathway is also inhibited, ultimately leading to VAL-4 and LFA-1 expression decrease and U937-HUVEC adhesion reduction. Inhibiting Cx43 expression in U937 monocytes with siRNA-Cx43, (a) Cx43, PKC-*α*, and NOX2 expressions are decreased (NC: negative control, *n* = 4, ∗*P* < 0.05 vs. control; ^#^*P* < 0.05 vs. the LPS group); (b) the level of ROS is decreased (NC: negative control, *n* = 5, ∗*P* < 0.05 vs. control; ^#^*P* < 0.05 vs. the LPS group); (c) VLA-4 and LFA-1 expressions are decreased (NC: negative control, *n* = 4, ∗*P* < 0.05 vs. control; ^#^*P* < 0.05 vs. the LPS group); (d) U937-HUVEC adhesion is decreased (NC: negative control, *n* = 5, ∗*P* < 0.05 vs. control; ^#^*P* < 0.05 vs. the LPS group). (e) Effects of dexmedetomidine on Cx43 expression in U937 monocytes (*n* = 4, ∗*P* < 0.05 vs. control; ^#^*P* < 0.05 vs. the LPS group; ^&^*P* < 0.05 vs. the 0.1 nM dexmedetomidine group).

**Figure 5:**
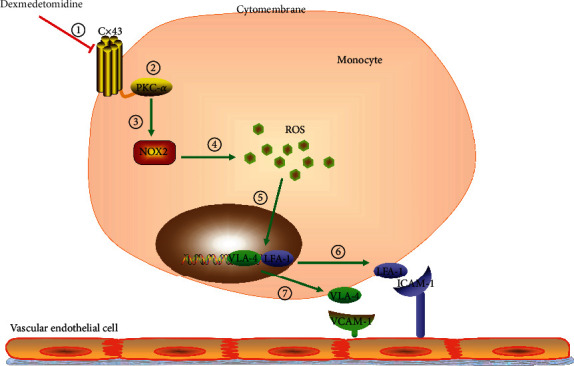
The possible mechanism of dexmedetomidine attenuating monocyte-endothelial adherence. (1) Dexmedetomidine inhibits Cx43 expression in U937 monocytes; (2) with the downregulation of Cx43, PKC-*α* interacting with Cx43 carboxyl-terminal domain is also decreased; (3) inhibiting PKC-*α* attenuates NOX2 expression; (4) ROS production is inhibited; (5) the decrease of ROS level reduces VLA-4 and LFA-1 expression in U937 monocytes; and (6, 7) VLA-4 and LFA-1 expressions are both attenuated, and they could not adhere to VCAM-1 and ICAM-1 on HUVECs.

As a transmembrane protein, it has been reported that the carboxyl-terminal domain of Cx43 can interact with some cellular signaling pathways, the most important one of which is just PKC [10, 12]. This kind of interaction provides the possibility that Cx43 expression on cell membrane can influence other signaling pathways in cytoplasm. We designed specific siRNA targeting to Cx43 expression. When Cx43 expression in U937 monocytes was knocked down, PKC-*α* and its downstream NOX2/ROS signaling pathway were also decreased significantly (Figures 4(a) and 4(b)). And simultaneously, with the downregulation of Cx43 expression in U937 monocytes, adhesion of molecule expression (VLA-4 and LFA-1) and U937-HUVEC adhesion were both attenuated (Figures 4(c) and 4(d)).  Figure 4(e) shows that dexmedetomidine (0.1 nM and 1 nM for 24 hours) alleviated LPS-induced Cx43 expression increase in U937 monocytes obviously. In summary, we concluded that dexmedetomidine application inhibited PKC-*α*/NOX2/ROS signaling pathway through downregulating Cx43 expression on cell membrane, resulting in the decrease of adhesion of molecule expression (VLA-4 and LFA-1) and U937-HUVEC adhesion (Figure 5).

## 4. Discussion

In present study, we found that dexmedetomidine, widely used in anesthesia and intensive care units, could significantly reduce U937-HUVEC adhesion when acting in U937 monocytes. The underlying mechanism was that dexmedetomidine, at its clinically relevant concentrations (0.1 nM and 1 nM for 24 hours), decreased Cx43 expression on cell membrane and PKC-*α* expression associated with the carboxyl-terminal domain of Cx43 protein. With the downregulation of PKC-*α*, the NOX2/ROS signaling pathway was inhibited, which resulted in the decrease of VLA-4 and LFA-1 expression (the ligands of important adhesion molecules VCAM-1 and ICAM-1). Ultimately, the U937-HUVEC adhesion was alleviated.

As a highly selective alpha-2 adrenoceptor agonist with sedative, analgesic, and anesthetic effects, dexmedetomidine is widely used for sedating patients in operation rooms or intensive care units [23, 24]. In these kinds of clinical settings, patients always suffer from high-level LPS and experience a long period of supine position. Monocytes will be easy to adhere to the inflamed vascular endothelial cells, aggravating vascular damage, or thrombosis. Through our present study, we found that when U937 monocytes were pretreated with dexmedetomidine, it could alleviate LPS-induced U937-HUVEC adhesion increase via downregulating Cx43 expression, providing a potent strategy against monocyte-endothelial adherence in clinic.

The role of Cx43 in monocyte-endothelial adherence is widely focused in recent years. A series of investigations, including ours showed that inhibiting Cx43 expression could attenuate monocyte-endothelial adherence [7, 9, 25]. Although the phenomenon that Cx43 could regulate monocyte-endothelial adherence has been observed, it is still unclear how Cx43 interacts with its downstream signaling pathways. When the extracellular environment changes, the proteins located on the surface of the cell membrane are always the first to be affected. Therefore, we observed that when exposed to LPS, the transmembrane protein, Cx43 expression in U937 monocytes was upregulated, which resulted in the increase of U937-HUVEC adhesion. With the downregulation of Cx43 expression in U937 monocytes, U937-HUVEC adhesion was attenuated. These results demonstrated that LPS-induced monocyte-endothelial adherence increase also started from acting on the cell membrane. According to the analysis of the transmembrane structure of Cx43 protein, we noticed that its carboxyl-terminal domain could interact with some cellular signaling pathways, the most important one of which was just PKC [9, 26]. This kind of interaction provided the possibility that Cx43 expression on cell membrane influenced other signaling pathways in cytoplasm. Our results also supported this hypothesis. In U937 monocytes, inhibiting Cx43 expression with specific siRNA, PKC-*α* expression, NOX2/ROS signaling pathway activity, and VLA-4 and LFA-1 expression were all decreased.

NADPH oxidases contain different subtypes, which are well known to be involved in signal transduction associated with inflammation and oxidative stress [27, 28]. In these subtypes, NOX2 abundantly expressing in monocytes is currently accepted as the main source of ROS [29, 30]. PKC activation also has been identified to play an important part in inflammation and oxidative stress [31, 32]. It has been reported that the cross talk between PKC and NOX could induce NOX-dependent ROS production [33]. In rat brain astrocytes, bradykinin could induce inflammation via activating PKC-*α*-mediated NOX2/ROS signaling pathway [34]. In lung epithelial A549 cells, resveratrol could also alleviate oxidative and inflammatory factors via inhibiting PKC-*α*/NOX2 signaling pathway [33]. In our present study, we also confirmed the relationship between PKC-*α* and NOX2 in U937 monocytes. With the downregulation of PKC-*α*, NOX2 expression and its related ROS production both decreased. We believed that the changes of Cx43 on cell membrane could regulate NOX2/ROS signaling pathway in cytoplasm through the interaction between Cx43 carboxyl-terminal domain and PKC-*α*, completing signal transduction from extracellular to intracellular.

ROS-mediated oxidative stress is thought to be closely related to all known cardiovascular diseases and also plays a central role in the process of monocyte-endothelial adherence [17, 35]. Multiple studies have shown that ROS can activate NF-*κ*B, JNK/SAPK, and p38 MAPK signaling pathways [36]. In vascular endothelial cells, ROS can result in the upregulation of adhesion of molecule expression significantly, increase monocyte-endothelial adherence, and ultimately atherosclerosis [37]. Rains et al. report that ROS can result in the increase of LFA-1 on monocytes [36]. And our investigation also demonstrates that scavenging ROS with NAC or inhibiting NOX2 with GSK2795039 can attenuate LPS-induced VLA-4 and LFA-1 expression in U937 monocytes and alleviate U937-HUVEC adhesion. This shows that inhibiting NOX2/ROS signaling pathway might be an effective strategy to reduce monocyte-endothelial adherence. In our present study, we first reported that Cx43 locating on cell membrane and PKC-*α* related with Cx43 carboxyl-terminal domain affected VLA-4 and LFA-1 expression in U937 monocytes via regulating NOX2/ROS signaling pathway. Dexmedetomidine could decrease VLA-4 and LFA-1 expression and U937-HUVEC adhesion via inhibiting Cx43 expression in U937 monocytes.

According to the results we have got in our investigation, we briefly summarize as follows (Figure 5): (1) Dexmedetomidine inhibits Cx43 expression in U937 monocytes; (2) with the downregulation of Cx43, PKC-*α* interacting with Cx43 carboxyl-terminal domain is also decreased; (3) inhibiting PKC-*α* attenuates NOX2 expression; (4) ROS production is reduced; (5) the decrease of ROS level reduces VLA-4 and LFA-1 expressions in U937 monocytes; and (6, 7) VLA-4 and LFA-1 expressions are both attenuated, and they could not adhere to VCAM-1 and ICAM-1 on HUVECs.

## 5. Conclusions

Patients undergoing major surgeries or remaining in intensive care units suffer high-level LPS and experience a long-term supine position [5, 6], which greatly increase the risk of monocyte-endothelial adherence and trigger inflammatory vascular injury. It is detrimental to the patient's recovery, especially for critical patients. Thus, it is very crucial to find out effective strategies to protect against monocyte-endothelial adherence. Dexmedetomidine is widely used for sedating patients in operation rooms or intensive care units [38, 39]. Through our present study, we found that dexmedetomidine could protect against monocyte-endothelial adherence effectively through regulating Cx43 and its related downstream PKC-*α*/NOX2/ROS signaling pathway. Therefore, rational use of dexmedetomidine for critical patients to avoid monocyte-endothelial adherence and its related vascular injuries should be highly valued by clinicians, especially by anesthesiologists and surgeons.

## Data Availability

All data generated or analyzed during this study are included in this published article.
